# Forceps for Extraction of Teeth

**Published:** 1854-01

**Authors:** 


### FORCEPS FOR EXTRACTION OF TEETH.

We find the Profession are becoming more and more particular in the style and adaptation of these useful instruments. Admitting that a beautiful instrument hurts as badly as an ugly one, yet certainly when beauty can be made consistent with utility, it is more agreeable and sightly to have deformity removed. These impressions have been forced upon us by the examination of a magnificent set of Forceps now for sale by Dr. J. M. Brown, corner 4th and Walnut streets. These were manufactured by Mr. H. R. Sherwood, of this city, and with their fine beaks, their curved handles, and highly polished appearance, look as if they would clasp a tooth in mere playful dalliance. And to still more captivate the eye, they are splendidly ornamented with jewels, which look like eyes sparkling with intelligence and saying, fear not, we never hurt. Here is evidently a combination of beauty and utility. These, in the case, are surrounded by magnificent dental minors, mouth glasses, and various articles of utility to the dentist.

But we wish especially to speak of some teeth—an improvement on the new style for Dr. Allen's continuous gum work. These are just received from Messrs. Jones, White & McCurdy, and in beauty of finish and coloring they are equal to the best they have ever sent to this market. The improvement in these teeth consist in the finish of the palital face and their natural and symmetrical body. The teeth are not so thick and clumsy as the first made of this pattern. Dr. Allen recently showed us two or three operations put up with these teeth, and finished in a little different style from any we had before seen. After the teeth are adjusted to their place on the platinum plate, and before the body is flown in about them, a little plaster is built up along the lingual base of the teeth—just enough to give a rounded and natural form to the lingual part of the denture—an impression is taken from this, and a thin platinum plate, stuck up to fit this, coming up and fastening to the platina wires of the teeth. When the teeth are soldered this plate is also soldered to its place, and gives a fine finish to the entire palital portion of the operation—leaving the crown of all the teeth uncovered by metal. A rim having been soldered around the labial border, the body and gum is put on and additional strength is secured. The teeth designed for this work are the best we have ever seen, and althongh costing a trifle more than the old style, yet, for operations in the mouth, we have always regarded that as economy which gets the best, irrespective of the price. Cheap dentistry and cheap materials are incompatible with good operations.

In addition to a fine lot of these teeth, Dr. Brown has just received an additional supply of other teeth from the same manufactory, which appear to have been gotten up with the same care and finish as the others.

